# TMC4 is a novel chloride channel involved in high-concentration salt taste sensation

**DOI:** 10.1186/s12576-021-00807-z

**Published:** 2021-08-25

**Authors:** Yoichi Kasahara, Masataka Narukawa, Yoshiro Ishimaru, Shinji Kanda, Chie Umatani, Yasunori Takayama, Makoto Tominaga, Yoshitaka Oka, Kaori Kondo, Takashi Kondo, Ayako Takeuchi, Takumi Misaka, Keiko Abe, Tomiko Asakura

**Affiliations:** 1 Department of Applied Biological Chemistry, Graduate School of Agricultural and Life Sciences, The University of Tokyo, 1-1-1 Yayoi, Bunkyo-ku, Tokyo, 113-8657 Japan; 2 Department of Food and Nutrition, Kyoto Women’s University, 35 Kitahiyoshicho Imakumano Higashiyama, Kyoto, 605-8501 Japan; 3 Department of Agricultural Chemistry, Meiji University, 1-1-1 Higashimita, Tama-ku, Kawasaki, Kanagawa 214-8571 Japan; 4 Department of Biological Sciences, Graduate School of Science, The University of Tokyo, 7-3-1, Hongo, Bunkyo-ku, Tokyo, 113-0033 Japan; 5 Division of Cell Signaling, National Institute for Physiological Sciences, National Institutes of Natural Sciences, 5-1 Aza-Higashiyama, Myodaijicho, Okazaki, Aichi 444-8787 Japan; 6 Thermal Biology Research Group, Exploratory Research Center On Life and Living Systems (ExCELLS), National Institutes of Natural Sciences, 5-1 Aza-Higashiyama, Myodaijicho, Okazaki, Aichi 444-8787 Japan; 7 Laboratory for Developmental Genetics, RIKEN-IMS, 1-7-22 Suehiro-cho, Tsurumi-ku, Yokohama, Kanagawa 230-0045 Japan; 8 Department of Integrative and Systems Physiology, Faculty of Medical Sciences, and Life Science Innovation Center, University of Fukui, Fukui, 910-1193 Japan; 9 Kanagawa Institute of Industrial Science and Technology (KISTEC), LiSE 4F C-4, 3-25-13 Tonomachi, Kawasaki-ku, Kawasaki, Kanagawa 210-0821 Japan

**Keywords:** Salty taste, Transmembrane channel-like protein, Chloride channel, Taste receptor, Taste signaling

## Abstract

“Salty taste” sensation is evoked when sodium and chloride ions are present together in the oral cavity. The presence of an epithelial cation channel that receives Na^+^ has previously been reported. However, no molecular entity involving Cl^−^ receptors has been elucidated. We report the strong expression of transmembrane channel-like 4 (TMC4) in the circumvallate and foliate papillae projected to the glossopharyngeal nerve, mediating a high-concentration of NaCl. Electrophysiological analysis using HEK293T cells revealed that TMC4 was a voltage-dependent Cl^−^ channel and the consequent currents were completely inhibited by NPPB, an anion channel blocker. TMC4 allowed permeation of organic anions including gluconate, but their current amplitudes at positive potentials were less than that of Cl^−^. *Tmc4*-deficient mice showed significantly weaker glossopharyngeal nerve response to high-concentration of NaCl than the wild-type littermates. These results indicated that TMC4 is a novel chloride channel that responds to high-concentration of NaCl.

**Supplementary Information:**

The online version contains supplementary material available at 10.1186/s12576-021-00807-z.

## Background

Recently, excessive salt intake has become a critical health problem worldwide [1]. Since saltiness is a basic taste that determines food quality, it is difficult to reduce the salt content in food, and there is no alternative for sodium chloride (NaCl). Prior to reducing salt intake, it is important to understand the mechanism underlying the perception of the “salty taste”.

Saltiness is evoked when both sodium and chloride ions are present in the oral cavity. Potassium chloride (KCl) and sodium sulphate (Na_2_SO_4_) also have a NaCl-like taste; however, it is far from the “authentic salt taste”. Hence, NaCl has a standard salty taste [2–4], which is currently the focus of public attention from sensory and nutritional points of view.

The mechanism of salt taste perception has been studied exclusively in rodents. Salt taste reception is initiated through the amiloride-sensitive and amiloride-insensitive pathways. Amiloride is an inhibitor of epithelial Na^+^ channel [5–8]. Amiloride-sensitive salt reception corresponds to low-concentration of salt, 100 mM or less, in the fungiform papillae (FuP) of the anterior tongue projecting to the chorda tympani nerve. Conversely, amiloride-insensitive salt reception corresponds to high-concentration of salt, 300 mM or more, in the circumvallate papillae (CvP) and foliate papillae (FoP) of the posterior tongue projecting to the glossopharyngeal nerve [9–11]. The epithelial Na^+^ channels (ENaC) α, β, and γ have been reported as the salt taste receptors for amiloride-sensitive pathways [5–11]. ENaC α-deficient mice have been shown to lose the low-concentration salt taste response that is transmitted through the chorda tympani nerve [6, 8, 9]. However, there are no well-defined molecules that have been shown to function as high-concentration salt taste receptors. Cation channels have been noted with respect to salt taste receptors, as evidenced by the reports that some cation channels are expressed in taste cells [12].

As for the anion, the taste responses for Na-gluconate and Na-acetate are lower than that for NaCl. The difference in salt taste intensity according to anion types is called "anion effects" [13–15]. In particular, the glossopharyngeal nerve response related to high-concentration of salt has been reported to be strongly influenced by anions [16]. The anion effect phenomenon was hypothesized as the anion-mediated inhibition of the depolarization of taste cells, in that, the effects depend on the size of the anion [13, 15, 17]. In contrast, no reports are available on the direct involvement of anion channels in salt taste reception on taste cells. Recently, Lewandowski et al. [18] and Roebber et al., [19] renewed past hypotheses about anion effect. They suggested that some molecules expressed in taste cells may mediate anion signals in association with salty taste. However, these molecules are yet to be elucidated. Therefore, there are many unresolved questions on high-concentration salt taste signal transduction and counter-ion dependency.

In this study, we aimed to resolve the questions pertaining to high-concentration salt taste reception, with particular focus on transmembrane channel-like 4 (TMC4). Using electrophysiological analysis, mouse TMC4 (mTMC4) was defined as a novel voltage-dependent Cl^−^ channel. It was found that the glossopharyngeal nerve response of *Tmc4*-deficient mice was significantly suppressed compared to that of their wild-type (WT) littermates. These results suggested that TMC4 was involved in high-concentration salt taste sensation. The findings of this study provide a clue to resolving a long-standing question regarding the role of Cl^−^ in salt taste perception.

## Methods

### RNA-sequencing analysis

Total RNA was extracted from the CvP or Epi of C57BL/6J mice (*n* = 20 or more) and pooled for each (CLEA, Tokyo, Japan). Poly (A)^+^ RNA was engineered into the DNA library using a TruSeq RNA sample preparation kit (Illumina, CA, USA). Sequence data were generated on a Genome analyzer IIx (Illumina). Per sample, 3 × 10^7^ reads (75 bp, single-ended) were obtained, and mapped using Bowtie 0.12.7 on the mouse genome (data assembly Jul. 2007, NCBI37/mm9). The expression of protein coding genes was analysed using ERANGE software to obtain reads per kilobase (exon model) per million mapped reads (RPKM) values. The steps after RNA extraction were carried out by Takara Bio, Shiga Japan.

### cDNA clones

m*Tmc4* cDNA (Reference sequence (Refseq) No. NM_181820.2) fragments were amplified from the CvP cDNA of C57BL/6J mice using the *Tmc4* forward primer (5'-GAATTCATGGAAGCCTGGGGCCAGTC-3') and the *Tmc4* reverse primer (5'-GCGGCCGCTCAGGAAGTTCCATTCCTTGAG-3'). We purchased cDNAs for mANO1 (Refseq No. BC062959) and hTMC4 (Refseq No. BC025323) from Dharmacon (Lafayette, CO, USA). The coding regions of the channels were cloned into EcoRI and Not I sites of pBluescript SK (Stratagene, USA) for ISH or into EcoRI and NotI sites of pIRES2-AcGFP1 (Takara Bio, USA), where the internal ribosome entry site and GFP-coding region were excised for the patch clamp experiment. All constructs were confirmed by nucleotide sequencing (Eurofins Genomics, Japan).

### Reverse transcription–polymerase chain reaction (RT-PCR)

cDNA fragments were obtained by RT-PCR using total RNA from the circumvallate papillae (CvP) of C57BL/6J mice (wild type, male, 12–30 weeks old). The epithelial tissue surrounding the taste buds was included in the CvP sample. A portion of the cDNA was used for standard PCR to detect *Tmc1–8 and* a representative taste marker, *transient receptor potential cation channel subfamily M member 5* (*Trpm5*). PCR primers were designed as follows: *Tmc1* (product size: 497 bp): forward primer, 5′‐CTTGAGACCAAAGAGGAAACGGA‐3′; reverse primer, 5′‐GAACCATGTTGACGCCGTACA‐3′. *Tmc2* (product size: 395 bp): forward primer, 5′‐CTCTGTTTGAAACCATCGCT‐3′; reverse primer, 5′‐CCAGCAGTGATTCATGAACC‐3′. *Tmc3* (product size: 349 bp): forward primer, 5′‐GTAGAAGAGACAAGCTTTCTGAC‐3′; reverse primer, 5′‐CATTGAATGCTGGCAGACAC‐3′. *Tmc4* (product size: 453 bp): forward primer, 5′‐GGACGCTGAAGAAAATTGGG‐3′; reverse primer, 5′‐CTGAGAACACTCGGTGACTG‐3′. *Tmc5* (product size: 393 bp)*:* forward primer, 5′‐CCAGCCAAGGACCATGCAAG‐3′; reverse primer, 5′‐ACCGTGTCCCCAAAATAACCC‐3′*. Tmc6* (product size: 469 bp): forward primer, 5′‐TGGTGGTCAGTGTCCTTAACCTG‐3′; reverse primer, 5′‐ATCAGGCTGGCCTTCTTGATGTG‐3′. *Tmc7* (product size: 312 bp): forward primer, 5′‐CAAGAGCTGCCAAGCTATCGG‐3′; reverse primer, 5′‐CAGTTCCAGGTGGGTTGTCATC-3′. *Tmc8* (product size: 313 bp): forward primer, 5′‐CTTCTGGGCCACCAAGTACTC‐3′; reverse primer, 5′‐GTTCTCCCAGCACTGGTAGTC‐3′. *Trpm5* (product size: 455 bp): forward primer, 5′‐CTGATCGCCATGTTCAGCTA‐3′; reverse primer, 5′‐ATGACGGATACACTGGCTCC‐3′. The probe template for *Tmc4* gene (NM_181820.2, probe region: 386–838) were obtained by RT‐PCR.

### In situ hybridization (ISH)

The chromogenic ISH was performed as described previously [20]. In brief, freshly frozen sections (7 µm thickness) of the CvP and FoP were fixed in PBS containing 4% paraformaldehyde and hybridized with a digoxigenin (DIG)-labelled *Tmc4* anti-sense probe (Roche Diagnostics, Switzerland). The probe was detected by alkaline phosphatase (AP)-conjugated anti-DIG antibody (Roche Diagnostics, Switzerland) and visualized by 4-nitro blue tetrazolium chloride (NBT)/5-bromo-4-chloro-3-indolyl-phosphate (BCIP) as a chromogenic substrate. A DP71 digital camera (Olympus, Tokyo, Japan) was used for signal detection.

For the fluorescence and chromogenic double labelling, fresh frozen sections (10 µm thickness) of CvP were fixed in PBS containing 4% paraformaldehyde and treated with 1 μg/ml proteinase K for 5 min at 30 °C. The sections were acetylated with 0.25% acetic anhydrate in 10 mM triethanolamine for 10 min at room temperature. After pre-hybridization, the sections were hybridized with DIG-labelled Tmc4 anti-sense probe and FITC-labelled taste cell marker anti-sense probes at 58 °C for 1–2 days. The sections were first incubated with peroxidase-conjugated anti-DIG antibody (1:100; Roche Diagnostics) for 1 h at room temperature. The sections were treated with TSA biotin system (1:50; Perkin Elmer, USA), and then incubated with streptavidin-conjugated Alexa Fluor 488 (1:300; Invitrogen) for 30 min at room temperature. The fluorescent images were taken by a BX51 microscope equipped with a DP71 digital camera (Olympus, Japan). Next, the sections were incubated with AP-conjugated-anti FITC antibody (1:500; Roche) for 1 h at room temperature, and the signals were developed using NBT/BCIP for 10–18 h at room temperature. The fluorescence and chromogenic images were overlapped using Photoshop Elements 14 (Adobe Systems, USA).

### Double-fluorescent immunostaining

Double-fluorescent immunostaining using rabbit anti-gustducin (1:500, Santa Cruz Biotechnology, Dallas, TX) and goat anti-Kcnq1 (1:1000, Santa Cruz Biotechnology), or rabbit anti-Plcβ2 (1:500, Santa Cruz Biotechnology) and goat anti-Car4 antibodies (1:500; R&D systems, Minneapolis, MN) was performed as described previously [21]. Briefly, B6 and Tmc4 KO mice were killed by an overdose of intraperitoneal sodium pentobarbital and transcardially perfused with 4% paraformaldehyde (PFA) in PBS. Tongue were dissected, post-fixed in 4% PFA/PBS and substituted by 20% sucrose/PBS. CvP were removed from the tongue, and then frozen in an optimal cutting temperature compound (Sakura Finetek, Tokyo, Japan). The CvP were sectioned at 7-µm with a cryostat. The CvP sections were washed with PBS and incubated in antigen retrieval solution for 20 min at 80 °C (Dako Target Retrieval Solution, pH 9, Agilent Technologies, Santa Clara, CA). The slides were then blocked with PBS containing Blocking One Histo (Nacalai tesque, Kyoto, Japan) and incubated with primary antibodies at 4 °C for overnight followed by incubation with Alexa Fluor 488-conjugated donkey anti-rabbit IgG and Alexa Fluor 555-conjugated donkey anti-goat IgG (1:500, Thermo Fisher Scientific) for an hour. The sections were then rinsed with PBS and mounted with Fluoromount.

### Transfection and patch clamp recording

HEK 293T cells (1.2 × 10^6^ cells per 35 mm culture dish) were transfected with 1 μg of plasmid encoding the channel (mTMC4, mANO1, or hTMC4), and 0.1 μg of p-EGFP-N1 (Addgene, Cambridge, MA, USA) using Lipofectamine LTX (Invitrogen, CA, USA). The cells were cultured on 18-mm diameter coverslips (Matsunami glass) in six-well plates and subjected to a whole-cell patch clamp experiment 24 to 36 h after transfection. The cells on the coverslips were placed in an RC-40LP bath chamber (Warner Instruments, Hamden, CT, USA) and observed under an IX-73 inverted microscope (Olympus, Tokyo, Japan). The cells exhibiting EGFP fluorescence were used for the experiment. Membrane current was recorded by an Axopatch 200B amplifier (Molecular Devices, San Jose, CA, USA) with a 5 kHz low-pass filter, digitized with a Digidata 1550 (Axon Instruments, Union City, CA, USA), and processed with pCLAMP 10.2 (Axon Instruments). The glass electrode was made from GD1.5 glass capillary tubes (Narishige, Tokyo, Japan) using a P-97/IVF micropipette puller (Sutter Instruments, Novato, CA, USA) to achieve a resistance of 3–5 MΩ. The series resistance was 6–10 MΩ and the correction of the capacitive component was performed at 10–20 pF. The bath solution was perfused at approximately 3500 μl/min and the whole apparatus was maintained at approximately 25 °C. For the halide ions experiment, a glass syringe needle MF28G-5 (World Precision Instruments, Sarasota, FL, USA) was used for perfusion. The cells were maintained at − 60 mV, with 10 mV step pulses of 400 ms applied from − 100 to + 100 mV, or ramp pulses from − 100 to + 100 mV of 300 ms every 5 s. The compositions of the pipette and the bath solutions are shown in Additional file 1:Table S1 and Table S2. We used the Clampex 10.2 (Axon Instruments) software to calculate and compensate for a liquid junction potential. The pH of the pipette and bath solutions was adjusted to pH 7.2 and 7.4 with NMDG-OH, respectively. The osmotic pressures of the pipette and both solutions were adjusted to approximately 270 and 295 mOsmol/kg, respectively. The free Ca^2+^ concentration was estimated using the MAXC program (Stanford University, Stanford, CA, USA) to obtain the concentration of 500 nM.

### Construction of a *Tmc4*-deficient mouse

*Tmc4*-deficient (*Tmc4* KO) C57BL/6J mice were generated by the Institute of Immunology Co., LTD. (Japan) using the transcription activator-like effector nucleases (TALEN) method (Additional file 1: Fig. S1). The founder mice were back-crossed and the frame shift mutations in the *Tmc4* gene were identified by direct sequencing of the genomic fragment from tails. The fragment was amplified using the primer set, 5'-GTCTGGCTGCCATGAAGTTTG-3' and 5'-CTGATATTAGGGACCCGTTTCCTG-3'. Two lines, delta 10 and 28, carrying frame shift mutations, were established and a sufficient number of litters were obtained. The lines were kept as heterozygotes and in-crossed to produce homozygote offspring. The mRNA of the *Tmc4* KO mice from the CvP was checked using PCR, which confirmed the deletion of the genome editing sequence.

### Maintenance of animals

All animal experiments were approved by the Animal Care and Use Committee at The University of Tokyo (Approval No. P17-034). We performed all animal experiments in accordance with relevant guidelines at the Committee of The University of Tokyo. The male offspring of line 10 were subjected to gustatory nerve recording and to the brief access test. Mice of line 28 were also subjected to gustatory nerve recording. We confirmed that there was no difference in taste preference between delta 10 and delta 28 lines by two bottle tests. Mice were fed a normal chow diet and housed at a constant temperature of 22 ± 1 °C under a 12-h light–dark cycle (lights on at 8 am). WT and *Tmc4* KO male mice (8–15 weeks old) were provided an AIN93-based powder diet and water ad libitum. Food and water intake was measured for 1 week. The mice were then transferred to metabolic cages (SN-783-0, Shinano, Japan) to obtain 24 h urine samples before killing for blood collection from the hepatic vein. All biochemical parameters were analysed by Nagahama Life Science Laboratory (Tokyo, Japan).

### Gustatory nerve recording

Gustatory nerve responses were measured in WT and *Tmc4* KO male mice of litters. Specifically, the glossopharyngeal nerve (*n* = 7 for WT and *n* = 6 for KO) and the chorda tympani nerve (*n* = 6 for WT and *n* = 5 for KO) were tested. Whole gustatory nerve responses from the glossopharyngeal and chorda tympani nerves were obtained as described previously [20, 22]. Briefly, the chorda tympani and glossopharyngeal nerves were exposed under anaesthesia. The exposed nerves were placed on a platinum wire electrode. An indifferent electrode was positioned near the wound. The whole-nerve activities were amplified 1000 times using a DAM80 amplifier (World Precision Instruments) and monitored on an oscilloscope. The detected impulses were integrated with a time constant of 1.0 s. The integrated nerve signal was sampled with a Power-Lab 4/30 analogue-to-digital converter (AD Instruments, Dunedin, New Zealand) for recording and data analysis on a computer using LabChart 8.0 software (AD Instruments). Tastants were applied for 30 s followed by rinsing with deionized water for > 30 s. Each tastant concentration was presented at least twice, and the mean whole-nerve response was calculated. The magnitude of the whole-nerve response was measured as the height of the integrated response from the baseline (before stimulation) to approximately 5 s after the onset of stimulation to avoid the tactile effects of the stimuli. For the glossopharyngeal nerve, the tastants used were 30–300 mM NaCl, 50–300 mM KCl, 300 and 500 mM sucrose, 100 and 300 mM MSG + 0.5 mM IMP, 1 and 3 mM denatonium, and 10 and 30 mM citric acid. For the chorda tympani nerve, the tastants used were 30–300 mM NaCl, 50–300 mM KCl, 100 and 300 mM sucrose, 30 and 100 mM MSG + 0.5 mM IMP, 10 and 30 mM denatonium, 10 and 30 mM citric acid, and 300 mM NaCl + 30 μM amiloride. In the case of NPPB application, the nerve responses to the salts were normalized by the responses to 100 mM NH_4_Cl to minimize individual fluctuations. The inhibitors, NPPB (100 μM) was included in 100 and 300 mM NaCl solutions. In the case of WT/*Tmc4* KO comparison, the nerve responses were not normalized by NH_4_Cl responses, but were represented as integrated impulses (the originally recorded nerve signals).

### Brief access test

Male mice of litters (*n* = 9 for WT and *n* = 7 for *Tmc4* KO) were tested in a special cage (Neuroscience, Tokyo, Japan) and their behaviour was recorded by a VSK0780 digital camera (Panasonic, Japan). The recorded data were replayed at quarter speed so that licking number could be counted visually. Each sample was blinded to researchers. Lick numbers were counted for 5 s initially. In the case of high-concentrations of NaCl and KCl, as well as citric acid, denatonium (Fig. 4A, B and D), mice were deprived of water for 23 h before the test. In the case of sucrose and MSG + IMP (Fig. 4D), mice were deprived of water for 4 h and 6 h, respectively, before the test. In the case of low-concentration NaCl (Fig. 4C), mice were injected with furosemide (50 mg/kg; Tokyo Chemical Industry, Japan) and given a low sodium diet 24 h before the test, as described previously [9].

The lick ratio for the tastants was calculated as the number of licks of tastant/the number of licks of water. The tastants used were 30–1000 mM NaCl (for high salt), 2–200 mM NaCl (for low salt), 30–1000 mM KCl (for high salt), 300 mM sucrose (for sweetness), 300 mM MSG + 0.5 mM IMP (for umami), 10 mM denatonium (for bitterness), 30 mM citric acid (for sourness).

### Mathematical modelling and simulation

The basic frame of the taste bud cell model was adapted from the model by Kimura et al. [23]. The cell model was newly implemented with a TMC4 model and modified so that it can better reproduce various experimental data shown in Kimura et al. [23] and Ma et al. [24]. The detailed description of the model is presented in Additional file 2.

### Statistical analysis

The data are represented as mean ± standard error of the mean (SEM). Statistical analyses were performed on GraphPad Prism 6 software (GraphPad Software, San Diego, CA, USA). In the patch clamp recordings, significant differences were identified using one-way analysis of variance (ANOVA) followed by post hoc Tukey–Kramer tests using JMP software (SAS, Tokyo, Japan). In the gustatory nerve recordings, significant differences were identified using Welch’s *t*-test and two-way ANOVA followed by post hoc Dunnett’s test or Bonferroni correction. In the brief access test, significant differences were evaluated using Welch’s *t*-test and two-way ANOVA followed by a post hoc Bonferroni test. For all analyses, significance was inferred where corrected *p* < 0.05. The asterisks (*, **, ****) indicate statistical differences: *p* < 0.05, 0.01 and 0.0001, respectively.

## Results

### *Tmc4* was expressed in taste bud cells of the posterior tongue

**Fig. 1:**
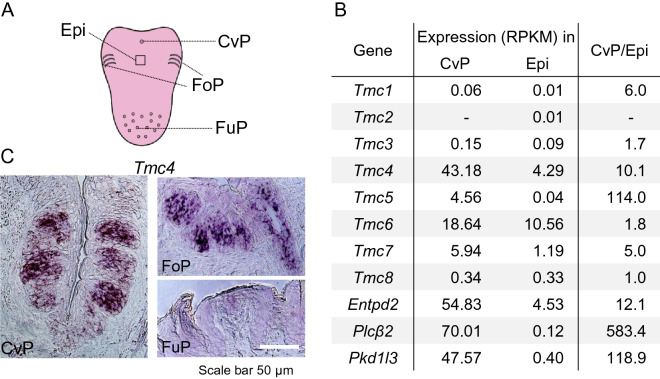
The *Tmc4* gene is predominantly expressed in the taste buds located at the posterior tongue. **A** Location of the circumvallate papillae (CvP), the foliate papillae (FoP), the fungi form papillae (FuP), and the epithelium (Epi) on the mouse tongue. **B** Expression of *Tmc* family genes in the CvP, Epi and specific expression level in the CvP (CvP/Epi). The data from next generation sequencing are represented as reads per kilobase (exon model) per million mapped reads (RPKM). The data for ectonucleoside triphosphate diphosphohydrolase 2 (*Entpd2*), phospholipase C beta 2 (*Plcβ2*), and polycystic kidney disease 1 like 3 (*Pkd1l3*) are indicated as representative taste cell markers. **C** In situ hybridization of *Tmc4* mRNA is specifically detected in the CvP and FoP, and minimally in the FuP

Previous work indicated that high-concentration of salt are expected to be received in the circumvallate papillae (CvP), which is innervated by the glossopharyngeal nerve [7, 9–11]. We therefore extracted total RNA from the CvP and the surrounding epithelia (Epi) to compare global mRNA expression in each tissue using next generation sequence (NGS) analysis. Genes specifically expressed at higher levels in the CvP than in the Epi, based on reads per kilobase (exon model) per million mapped reads (RPKM) were extracted (Fig. 1A). These were limited to genes with a CvP > 10 and CvP/Epi RPKM ratio > 3. As a result, 1120 candidate high-concentration salt receptor genes were identified. With the idea of narrowing this field, we noted that Chatzigeorgiou et al. [25] reported that one member of the TMC family functions as a NaCl sensor in nematodes. We therefore examined the expression of the mammalian TMC family in the taste papillae of the posterior tongue. The mouse TMC family is composed of eight members, named TMC1 through TMC8. Mammalian TMC families are distantly related in sequence to the anoctamin (ANO) families, some of which are Cl^−^ channels [26]. We found only three TMC molecules, *Tmc4*, *Tmc5*, and *Tmc7* in the above-mentioned 1120 genes, and only *Tmc4* was highly expressed in the CvP at levels close to those of known taste-transducing molecules, such as *Entpd2*, *Plcβ2*, and *Pkd1l3* (Fig. 1B and Additional file 1: Fig S2A). In our chromogenic in situ hybridization (ISH) experiment, *Tmc4* had a relatively strong signal in the CvP as well as in the foliate papillae (FoP), but was scarcely detected in the fungiform papillae (FuP) (Fig. 1C). We also found that Tmc4 was broadly expressed in taste cells of the CvP using some taste cell markers (Additional file 1: Fig S2B). These data motivated us to analyse TMC4 as a candidate salt taste perception molecule.

### TMC4 was a voltage-dependent chloride channel

**Fig. 2:**
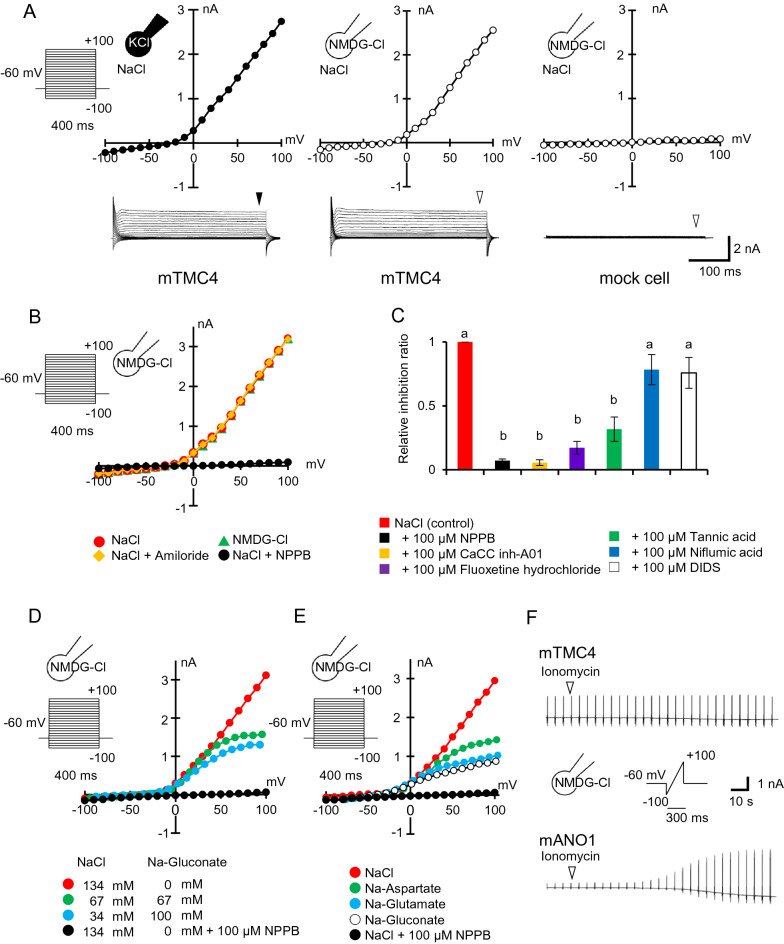
TMC4 functions as a novel anion channel. **A** Representative current–voltage (I-V) relationship of the currents by step pulses with KCl (left) or *N*-Methyl-d-glucamine (NMDG)-Cl (centre) pipette solution in human embryonic kidney (HEK) 293T cells expressing mouse TMC4. Right panel shows the response of mock cells as a negative control. Open and filled arrowheads show the points of data selection from raw trace shown below. **B** Representative I–V relationship of the currents by step pulses under different bath solutions in cation. The current observed is not affected by the bath application of NMDG-Cl, NaCl, or amiloride (epithelial sodium channel inhibitor), but is significantly reduced by the anion channel inhibitor, 5-nitro-2-(3-phenylpropylamino) benzoic acid (NPPB). **C** Effect of several anion channel inhibitors. Relative inhibition ratio calculated using the formula *I*_blocker_/*I*_control_ at + 60 mV. Ca^2+^-activated chloride channel inhibitor (CaCC inh)-A01: ANO1/2 inhibitor, tannic acid: cystic fibrosis transmembrane conductance regulator (CFTR) and CaCC inh, niflumic acid: CaCC inh, and 4,4′-diisothiocyanatostilbene-2, 2′-disulfonic (DIDS): anion exchanger inhibitor. Different letters show significant differences, detected by Tukey–Kramer test (*n* = 4 or more for each, *p* < 0.05). **D** Representative I–V curves by step pulses with different extracellular Cl^−^ concentrations through replacement with gluconate. **E** Representative I–V curves by step pulses with bath solution that has completely replaced a chloride ion with an organic anion. **F** Effects of increase in intracellular Ca^2+^ concentrations by ionomycin on mTMC4 (upper) or mANO1 (lower) -mediated currents. Open arrow heads indicate the starting point for perfusion of the bath solution containing ionomycin. Bath and pipette solution components and calculation procedures are shown in the Materials and methods section, Additional file 1: Table S1 and Table S2

To elucidate the properties of TMC4 channel, we used a whole-cell patch clamp technique with a heterologous expression system. Human embryonic kidney (HEK) 293T cells transfected with mTMC4 expression plasmid exhibited significant outward currents at positive potentials regardless of intracellular cations (K or *N*-methyl-d-glucamine, NMDG) while inward currents at negative potentials were very small in both conditions (Fig. 2A left and centre panels). Reversal potentials (Er) were nearly equal (mean ± SEM, *n* = 4 for each; − 27.6 ± 0.9 mV for KCl and − 25.6 ± 4.0 mV for NMDG-Cl). On the other hand, the mock cells without expressed mTMC4 exhibited no such currents (Fig. 2A right panel). Next, we examined the effect of extracellular cation composition. We found that the currents were independent of extracellular cations (NMDG-Cl, shown as green triangle in Fig. 2B) with a pipette solution of NMDG-Cl. The currents were inhibited by the anion channel inhibitor, 5-nitro-2-(3-phenylpropylamino) benzoic acid (NPPB, shown as black circle in Fig. 2B, [27], but not by amiloride (yellow rhombus in Fig. 2B). These results indicated that mTMC4 was neither a cation channel nor sensitive to amiloride (Fig. 2A, B). Then, several inhibitors of Cl^−^ and anion channels were examined [28–31]. mTMC4-mediated currents were strongly inhibited by the NPPB or Ca^2+^-activated chloride channel inhibitor, CaCC(inh)-A01 [29] (Fig. 2C). Fluoxetine hydrochloride and tannic acid also inhibited mTMC4-mediated currents although less effective with 100 μM, while no such inhibition was observed with niflumic acid or 4,4′-diisothiocyanatostilbene-2, 2′-disulfonic (DIDS) (Fig. 2C).

To confirm whether or not mTMC4 exhibits anion permeability, the Cl^−^ concentrations of the bath solution were decreased stepwise or the extracellular Cl^−^ ions were substituted with several organic anions (Fig. 2D, E). Surprisingly, I–V curves showed no apparent changes in reversal potentials although it was difficult to determine the precise reversal potentials partly because of small inward currents at negative potentials. While current amplitudes at positive potentials became smaller as the reduction in extracellular Cl^−^ concentrations or replacement with different anions using a pipette solution of NMDG-Cl. It is of note that the mTMC4-mediated outward currents did not disappear even when the bath solution was completely substituted with Na-gluconate, with a larger ion size than halide. These results suggested that mTMC4 functions as an anion channel with a large pore.

In view of previous evidence that TMC4 is distantly related to the ANO1 channel as a CaCC [26, 32], and that mTMC4-mediated currents were inhibited by CaCC(inh)-A01 (Fig. 2C), we examined the Ca^2+^ sensitivity of mTMC4. In contrast to mouse ANO1 (mANO1), the channel activity of mTMC4 did not depend on intracellular Ca^2+^ (Fig. 2F).

Next, we performed similar experiments on the human orthologue of mTMC4. We found that human TMC4 (hTMC4) also functions as a Cl^−^ channel with a similar voltage dependency and is inhibited by NPPB as seen in the mouse channel (Additional file 1: Fig S3). These results indicated that the function of mTMC4 was conserved in humans.

### Effect of NPPB on the taste nerve responses to salty taste

TMC4-mediated currents were strongly inhibited by NPPB in the in vitro response (Fig. 2). To obtain information about the effect of NPPB in the in vivo response to salty taste that Cl^−^ is involved in, we conducted taste nerve recordings with wild-type C57BL/6J mice (Additional file 1: Fig S4). The simultaneous application of 100 μM NPPB led to significant decrease of the glossopharyngeal nerve response at 300 mM NaCl while the same treatment had no effect on the chorda tympani nerve response. This indicates that high salt taste reception requires Cl^−^ perception.

### TMC4 functions in high-concentration salt taste transduction

**Fig. 3:**
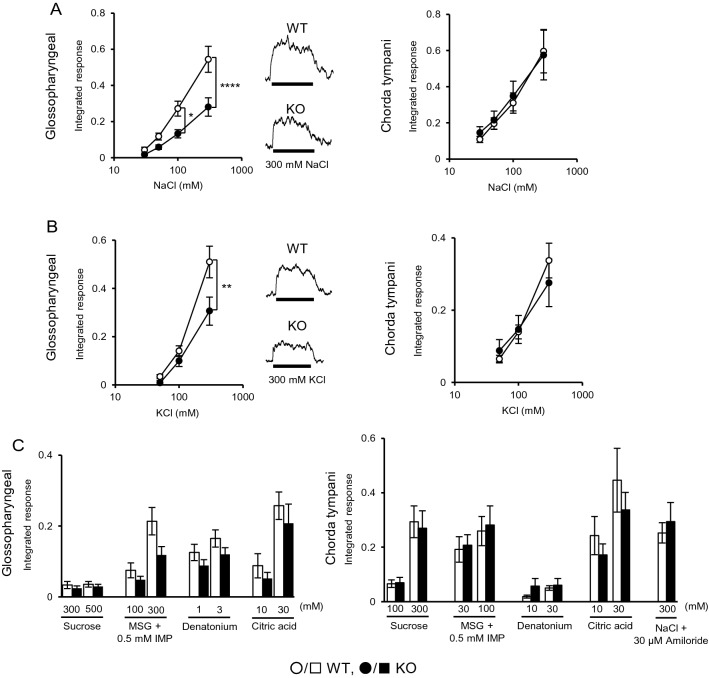
Loss of TMC4 specifically affects the glossopharyngeal nerve response to high NaCl and KCl. **A**, **B** Integral taste nerve response of wild-type (WT) or *Tmc4* knock-out (KO) mice to NaCl and KCl solutions (30, 50, 100, and 300 mM). **C** Responses to the other taste substances: sucrose for sweet, a mixture of monosodium glutamate (MSG) and inosine monophosphate (IMP) for umami, citric acid for sour, denatonium for bitter and amiloride for epithelial sodium channel blocker. The asterisks (*, **, ****) indicate statistical differences: *p* < 0.05, 0.01, and 0.0001, respectively. Significance was evaluated using two-way ANOVA with Bonferroni correction (*n* = 6–7 for WT and *n* = 5–6 for KO)

To confirm that TMC4 was related to salt perception, we generated *Tmc4* knock-out (KO) mice (Additional file 1: Fig S1). The loss of function of TMC4 in vivo caused a significant decrease in the glossopharyngeal nerve response to 100 mM and 300 mM NaCl, but no change in the chorda tympani nerve response (Fig. 3A). The glossopharyngeal nerve response to 300 mM KCl was also significantly affected, but no change was observed in the chorda tympani nerve response (Fig. 3B). No significant difference was observed between KO and WT mice for the other basic taste substances in both the glossopharyngeal and chorda tympani nerves including the response to 300 mM NaCl containing 30 µM amiloride (Fig. 3C).

Since *Tmc4* was systemically deficient, we checked the growth rate of animals and their mineral content in the sera and in the urine of *Tmc4* KO mice and found that there was no significant difference in Na, K, Cl, Ca, and Mg between KO and WT mice (Additional file 1: Fig S5A, B, C and Table S3). In addition, we observed the expression of representative taste-related proteins in the CvP. Immunoreactivity to the relevant antibodies showed no apparent differences in protein expression patterns, and in morphology of taste cells, in both KO and WT mice (Additional file 1: Fig S5D). These results made us to investigate the behavioural phenotype of *Tmc4* KO mice.

**Fig. 4:**
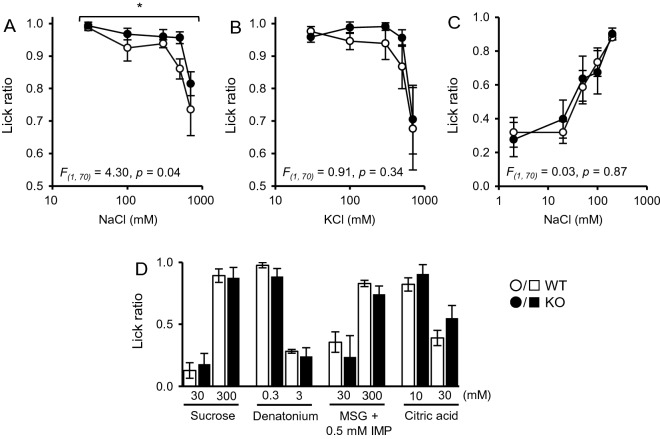
Licking behaviour of *Tmc4* knock-out (KO) mice with five basic tastes. **A**, **B** Lick ratio of wild-type (WT) or *Tmc4* KO mice for NaCl (**A**) and KCl (**B**) solutions (30, 100, 300, 500, and 700 mM) to water. **C** Attraction to NaCl solution (2, 20, 50, 100, and 200 mM) of mice treated with furosemide (50 mg/kg) and a low sodium diet. **D** Lick ratio for taste substance solutions to water: sucrose for sweet, a mixture of monosodium glutamate (MSG) and inosine monophosphate (IMP) for umami, citric acid for sour, denatonium for bitter. Data are expressed as the mean ± SEM. The asterisk (*) indicates statistical difference (*p* < 0.05). Significance was evaluated using two-way ANOVA (WT: *n* = 9, KO: *n* = 7)

*Tmc4* KO and WT mice were tested for their aversive behaviour to high-concentration salt solution by measuring the lick ratio to water. There was a slight difference in the lick ratio value for NaCl solutions between KO and WT mice (Fig. 4A; *F*_*(1, 70*)_ = 4.30, *p* = 0.04). However, no significant results were obtained with KCl solutions (Fig. 4B; *F*_*(1, 70)*_ = 0.91, *p* = 0.34), and there was preference for low-concentration salt solution after sodium-depletion treatment (Fig. 4C; *F*_*(1, 70)*_ = 0.03, *p* = 0.87). It was found that KO mice showed no change in the lick ratio for the other taste solutions (Fig. 4D).

## Discussion

In this study, mTMC4 was identified as a novel anion channel involved in high-concentration salt reception at taste bud cells of the posterior tongue. We discuss from the view points of the structure characterization and physiological properties.

### TMC4 was a voltage-dependent anion channel

TMC4 is a member of transmembrane channel-like (TMC) family having an expected eight to ten transmembrane-spanning domains and a relatively conserved sequence termed the TMC domain, which encompasses three transmembrane sequences near the C-terminus [26, 33, 34]. In mammals, eight molecules named TMC1 through TMC8 belong to this family [26, 34–36]. Of these eight members, TMC1 is well studied. A mutation in TMC1-expressing inner ear cells causes deafness [37, 38]. TMC2 is also involved in hearing through sound vibration-mediated Ca^2+^ mechanotransduction [39, 40]. Mouse TMC1 and TMC2 were already defined to be mechanosensitive cation channels using analysis of the homologues, green sea turtle (CmTMC1) and budgerigar (MuTMC2) [41]. As for the other TMC molecules, mutations in TMC6 (EVER1) and TMC8 (EVER2) are implicated in epidermodysplasia verruciformis [42]. TMC5 promotes prostate cancer cell proliferation [43] and TMC7 is upregulated in cases with onset of pancreatic carcinoma [44]. Thus, almost all genes belonging to the TMC family have a physiologically important role. However, the molecular characterization of TMC members has not been defined except for TMC1 and TMC2.

In this study, we revealed that TMC4 is an anion channel involved in salt taste perception. To the best of our knowledge, this is the first report of an anion channel belonging to the TMC family (Fig. 2). All three amino acids, G520, M521 and D672 important for mechanosensitive cation channel in TMC1 and TMC2 are not conserved in TMC4 [41]. Incidentally, because the structure of TMC members is similar to the ANO channels, TMC is assigned to the ANO superfamily [26, 33]. Of the ten mammalian ANO members, ANO1 through ANO10, ANO1 and ANO2 were reported to be CaCCs [26, 32, 45]. However, recently ANO4, ANO6, and ANO9 have been reported to be cation channels [46–48]. Considering the case of the ANO family, it is not particularly rare that anion and cation channels belong to the same family.

Besides, mTMC4 was inhibited by CaCC inh-A01, a specific blocker of ANO1 and ANO2 [29]. It was suggested that TMC4 had a common structure with ANO1 in the activation site. However, in contrast to mANO1, mTMC4 was activated without an increase of intracellular Ca^2+^. The Ca^2+^ binding site was reported to be N646, E650, E698, E701, E730, E734 in mANO1 [49–54]. Comparing the mTMC4 sequence with mANO1, only one residue, E698 in mANO1 and E513 in mTMC4, was conserved, but the other five residues were not. This implies that mTMC4 is insensitive to intracellular Ca^2+^.

Further, a distinct feature of mTMC4 was that anions with large ion sizes, such as gluconate ions, could also permeate through mTMC4 (Fig. 2D, E). It is a future interest to resolve structure basis at an atomic level for this broad anion permeability. Taking these data together, it was noted that mTMC4 is a novel voltage-dependent anion channel with unique features.

### Physiological function of TMC4 for salt taste reception as an anion channel

From the view point of physiological function, we first reported the possibility that TMC4 is involved in the perception of salty taste. In our study, *Tmc4* KO mice significantly reduced the glossopharyngeal nerve response to high-concentrations of salt. On the other hand, there was no significant difference in the responses of the chorda tympani nerves (Fig. 3). Behavioural responses are induced by integrated taste signals from both the glossopharyngeal and chorda tympani nerves. Therefore, the effect of *Tmc4* KO on behavioural response differs slightly from the glossopharyngeal nerve response (Fig. 4). ENaC, a sodium channel, was reported to respond to low-concentration of salt, while molecules responding to high-concentration of salt had not been reported, including molecules responding to Cl^−^. Counter salts anions affect high-concentration salt taste sensation at the level of the taste nerve response in rodents, and sensory evaluation in humans. This has been called the "anion effect". Theoretically, one feature of this effect is that salt taste intensity varies depending on anion size [2–4, 13–17]. Roebber et al. [19] have reported that taste cells have the apical transducer for anion, which is the key to elucidating the anion effect. However, the specific transducer was long unknown. Intriguingly, the TMC4-mediated current at the positive potential changed with the molecular size of the anion (Fig. 2E). It might explain the “anion effect” phenomenon where saltiness intensity decreases as the molecular size of the anion increases. In this respect, TMC4 proved to a prime example of the anion effect. Its identification and functional elucidation constitute the first proof at the molecular level that salt taste reception is dependent on Cl^−^ influx. Moreover, TMC4 may be related to salt taste signal transduction.

### Current status of salt taste reception on taste cells

There are two types of salt-responsive cells in mammalian taste buds: one responding to low-concentration of salt and the other to high-concentration of salt (Additional file 1: Table S4). Chandrashekar et al. [9] reported that low-concentration salt cells respond to 100 mM or higher concentration of NaCl or Na-gluconate and these responses are inhibited by amiloride. In addition, low-concentration salt cells do not respond to 500 mM NMDG-Cl or 500 mM KCl. The Na^+^-dependent nature of low-concentration salt cell function may correspond to the properties of the amiloride-sensitive ENaC channel, which is responsible for low-concentration salt taste reception. However, high-concentration of salt cells respond to 500 mM NaCl, 500 mM KCl, and 500 mM NMDG-Cl and these responses are not inhibited by amiloride. Lewandowski et al. [18] divided high-concentration of salt cells into two groups: one responding to 250 mM NaCl and 250 mM Na-gluconate equally in an anion-independent manner, and the other responding to 250 mM NaCl and 250 mM Na-gluconate (to a lesser extent) in an anion-dependent manner. These results suggested that the response of high-concentration of salt cells may be intimately associated with the behaviour of Cl^−^.

It is clear from our data that Cl^−^ influx through the TMC4 channel could only occur with a depolarization of taste cell (Fig. 2). There are several studies on the resting and action potentials (*V*_r_ and *V*_ac_) of taste bud cells. Ma et al. [24] reported that mouse taste bud cells exhibit *V*_r_ of − 65 mV and *V*_ac_ of + 50 mV, on average, in response to current stimulation. In a study by Chen et al. [55], V_r_ was approximately −3 3 mV and *V*_ac_ was approximately + 40 mV. These data suggested the existence of a molecular entity that triggers *V*_ac_ generation in taste bud cells. Actually, Cl^−^ influx is observed in many taste cells of the CvP in mice when depolarization occurs [56, 57]. In this context, Sukumaran's single cell transcriptome analysis is suggestive; they showed that TMC4 is co-expressed with voltage-gated Na^+^ channels (Scn2a1, Scn3a, and Scn9a) in 23 taste cells [58].

### Working hypothesis of TMC4 in high-concentration salt taste sensation

**Fig. 5:**
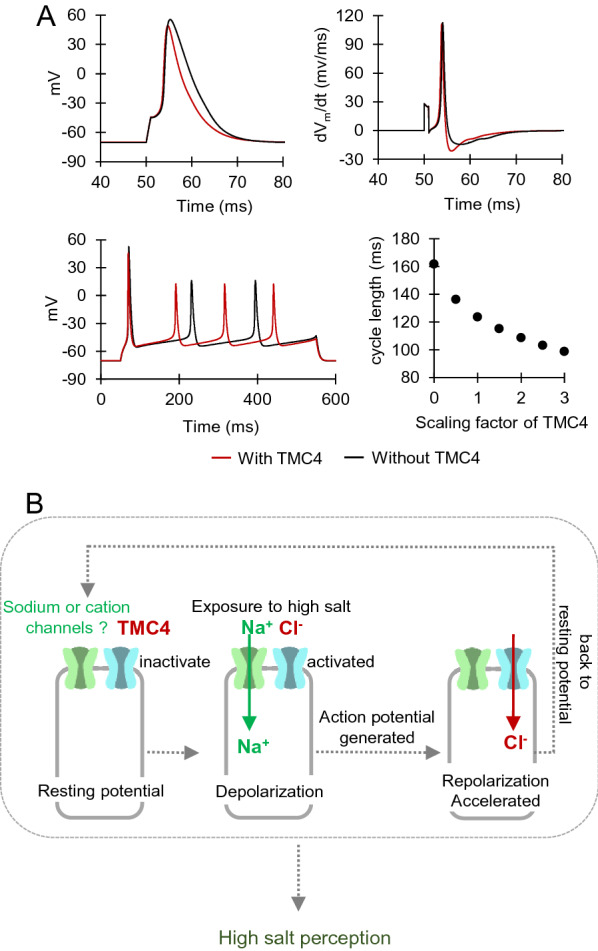
Working hypothesis of TMC4 role in high-concentration salt taste sensation. **A** Prediction of TMC4 role using taste cell model with (red) or without (black) TMC4. (left) Single action potentials evoked by 2 Hz stimulation with − 140 pA stimulation current (*I*_stim_) for 1 ms. (right) Time derivatives of the voltage (dV_m_/dt) during the action potentials. (bottom left) Trains of action potentials evoked by − 15 pA I_stim_ for 500 ms. (bottom right) Relationship between TMC4 expression level and cycle length. The conductance of TMC4 was amplified by 0 to 3.0, and then trains of action potentials were evoked by − 15 pA I_stim_ for 500 ms. The averaged cycle length between action potential peaks was plotted against multiplying factor of TMC4 conductance. In all these simulations, the model cell was held at − 70 mV by applying 10 pA holding current (*I*_hold_). [Na^+^]_o_ = 150 mM, [K^+^]_o_ = 5.4 mM, [Cl^−^]_o_ = 150 mM, [Na^+^]_i_ = 6 mM, [K^+^]_i_ = 140 mM, [Cl^−^]_i_ = 30 mM. **B** Schematic representation of salt taste reception involving TMC4. TMC4 is specifically expressed in high-concentration salt taste cells in the circumvallate papillae (CvP) and the foliate papillae (FoP). TMC4 is not activated at the resting membrane potential. Exposure of the taste cells to high-concentration of NaCl induce depolarization by Na^+^ influx through sodium or cation channels, which triggers action potential for salt taste signals. Furthermore, this depolarization activates TMC4. After depolarization, Cl^−^ influxes the taste cells through the TMC4 and helps the taste cells return to the resting potential. TMC4 consequently may accelerate the cycle of action potentials for salt taste signals. These processes facilitate neurotransmission via high-concentration salt taste cells, with the result that salt taste reception takes place through the glossopharyngeal nerve

Physiologically, a role of Cl^−^ influx to cell is regulation of membrane potential for hyperpolarization. It helps repolarize the cells to return to resting potential. Huang, et al. [59] reported that CACCs blocker, niflumic acid and NPPB, inhibited Cl^−^ influx, and delayed action potential in hippocampal pyramidal neurons. Based on these backgrounds, we simulated how Cl^−^ influx through TMC4 is involved in the action potentials for salt taste signals in taste cell of the CvP. The model cell predicted that TMC4 contributes to repolarization phase as revealed from larger negative dV_m_/dt value when TMC4 was incorporated, which shortens action potential duration (Fig. 5A upper). This resulted in shorter cycle lengths when the model cell was stimulated with longer depolarizing current pulse (I_stim_) (Fig. 5A lower left). The larger the expression level of TMC4 was, the shorter the cycle length between action potentials became (Fig. 5A lower right).

To summarize our data, we propose the working hypothesis to explain the role of TMC4, for high-concentration salt taste sensation (Fig. 5B). If the taste cells are stimulated by high concentrations of salt, taste cells are depolarized by Na^+^, which influx through sodium or cation channels. The depolarization triggers the action potentials and induces salt taste signals. Furthermore, this depolarization activates TMC4. When TMC4 is activated, extracellular Cl^−^ flows into the taste cells through TMC4 and returns the taste cells to the resting potential. Consequently, TMC4 might be involved in acceleration the cycle of action potentials for salt taste signals. To explore this model further, one potential line of future investigation is assessed as function of TMC4 in high-concentration salt sensation using isolated taste bud cells.

In many countries, the per-capita salt intake exceeds recommended levels [60]. Since the overconsumption of salt potentially affects the body’s liquid homeostasis [61] and the amiloride-insensitive pathway is dominant in salt taste perception in humans [62, 63], hTMC4 enhancers may be useful for the production of sodium-reduced foods, chemicals which are currently being screened. Future research on TMC4-mediated high-concentration salt taste sensation could therefore have an impact on human health as well as the food industry.

## Conclusion

Salty taste is evoked by the existence of both Na^+^ and Cl^−^. The present study shows that TMC4 is a novel voltage-dependent chloride channel involved in high-concentration salt taste. *Tmc4* was expressed in the CvP and FoP, and the glossopharyngeal nerve response to salty taste of *Tmc4*-deficient mice was significantly suppressed. Mathematical modelling and simulation revealed that the TMC4-mediated Cl^−^ current is involved in accelerating the cycle of action potentials. This is a new finding of the role of Cl^−^ involving salty taste acceptance.

### Supplementary Information

**Additional file 1: Figure S1.** *Tmc4* gene knock-out (KO) strategy. Mutations were introduced into exon 1 of the *Tmc4* locus by the transcription activator-like effector nucleases (TALEN) method. The DNA-binding sites of the TALENs are indicated by red and blue boxes. The start codon is shown in green characters. The broken line indicates deleted nucleotides. Two lines, delta 10 and 28 carrying frame shift mutations, were obtained and a sufficient number of litters were obtained from the delta 10 and 28 lines. These lines were back-crossed with C57BL/6J mice (wild-type: WT) to produce founder mice. **Figure S2.** Tmc4 gene is broadly expressed in the circumvallate papilla of taste buds located at the posterior tongue. **A** RT‐PCR of TMC family mRNAs (*Tmc1*–*Tmc8*) and *Trpm5* (taste cell marker) in the circumvallate papillae (CvP) of wild-type (WT) mice. **B** Co-localization of *Tmc4* (green signal) in the CvP of WT mice with ectonucleoside triphosphate diphosphohydrolase 2 (*Entpd2*) expressed in type 1 cells, phospholipase C beta 2 (*Plcβ2*) expressed in type 2 cells, or polycystic kidney disease 1 like 3 (*Pkd1l3*) expressed in type 3 cells, (purple signal) in the circumvallate papilla. For the negative control, sections were hybridized only with *Tmc4 sense* probe and detected by Alexa Fluor 488. **Figure S3.** Human TMC4 has the same properties as mouse TMC4. **A** Representative current–voltage (I-V) relationship of the currents by step pulses with KCl (left) or *N*-Methyl-d-glucamine (NMDG) -Cl (right) pipette solution in human embryonic kidney (HEK) 293 T cells expressing human TMC4 (hTMC4). **B** Representative I–V relationship of the currents by step pulses under different bath solutions in cation. The current observed is not affected by the bath application of NMDG-Cl, NaCl, or amiloride (epithelial sodium channel inhibitor), but is significantly reduced by the anion channel inhibitor, 5-nitro-2-(3-phenylpropylamino) benzoic acid (NPPB). Bath and pipette solution components and calculation procedures are shown in the Materials and Methods section, Table S1 and Table S2. **Figure S4.** Effect of NPPB on the taste nerve response to high NaCl in wild type mice. Wild type mice were treated with NaCl solutions (100 and 300 mM) with or without chloride channel inhibitor, 5-nitro-2-(3-phenylpropylamino) benzoic acid (NPPB). The integral response of the glossopharyngeal nerve to 300 mM NaCl was significantly suppressed by the simultaneous application of 100 μM NPPB. The chorda tympani response to 300 mM NaCl was not suppressed by 100 μM NPPB. The application of 100 μM NPPB itself did not cause any response in neither the glossopharyngeal nor chorda tympani nerves (Statistical differences evaluated by Welch's *t*-test. ^*^ indicate significant difference: *p* < 0.05. n = 5–6). **Figure S5.** Food intake, water intake, body weight and expression of taste marker molecules in the circumvallate papillae of wild-type (WT) and *Tmc4* knock-out (KO) mice, **A** and **B**. Comparison of food and water intake. **C** Comparison of body weight at the time of killing (8–15-week-old males). No significant differences were detected (WT: *n* = 6, KO: *n* = 10). **D** Comparison of expression of taste marker molecules in the circumvallate papillae. Representative double-fluorescence immunostaining images are shown. The immunostaining was examined using antibodies against the type2 cell marker (gustducin: green) and the taste bud cell marker, potassium voltage-gated channel subfamily Q member 1(Kcnq1: magenta), and phospholipase C beta 2 (Plcβ2: green) and Carbonic anhydrase 4(Car4: magenta). Scale bar is 50 μm. Merged fluorescence and differential interference contrast images were showed.

**Additional file 2.** Description of the Mathematical Model of a Taste Bud Cell.

## Data Availability

All sequence data used for this study have been deposited in NCBI’s Gene Expression Omnibus (GEO) with the accession number GSE175806.

## Abbreviations

TMC4: Transmembrane channel-like 4
CvP: Circumvallate papillae
FuP: Fungiform papillae
FoP: Foliate papillae
Epi: Epithelia
NMDG: *N*-Methyl-d-glucamine
NPPB: 5-Nitro-2-(3-phenylpropylamino) benzoic acid
DIDS: 4,4′-Diisothiocyanatostilbene-2, 2′-disulfonic
CaCC: Ca^2^^+^-activated chloride channel
ANO: Anoctamin
