# Supplementary material for: TMC4 is a novel chloride channel involved in high-concentration salt taste sensation
Source: J Physiol Sci. 2021 Aug 25;71:23. doi: 10.1186/s12576-021-00807-z (PMC10717410; doi:10.1186/s12576-021-00807-z)
Supplement: Supplementary file 1 — Additional file 1: Figure S1. Tmc4 gene knock-out (KO) strategy. Mutations were introduced into exon 1 of the Tmc4 locus by the transcription activator-like effector nucleases (TALEN) method. The DNA-binding sites of the TALENs are indicated by red and blue boxes. The start codon is shown in green characters. The broken line indicates deleted nucleotides. Two lines, delta 10 and 28 carrying frame shift mutations, were obtained and a sufficient number of litters were obtained from the delta 10 and 28 lines. These lines were back-crossed with C57BL/6J mice (wild-type: WT) to produce founder mice. Figure S2. Tmc4 gene is broadly expressed in the circumvallate papilla of taste buds located at the posterior tongue. A RT‐PCR of TMC family mRNAs (Tmc1–Tmc8) and Trpm5 (taste cell marker) in the circumvallate papillae (CvP) of wild-type (WT) mice. B Co-localization of Tmc4 (green signal) in the CvP of WT mice with ectonucleoside triphosphate diphosphohydrolase 2 (Entpd2) expressed in type 1 cells, phospholipase C beta 2 (Plcβ2) expressed in type 2 cells, or polycystic kidney disease 1 like 3 (Pkd1l3) expressed in type 3 cells, (purple signal) in the circumvallate papilla. For the negative control, sections were hybridized only with Tmc4 sense probe and detected by Alexa Fluor 488. Figure S3. Human TMC4 has the same properties as mouse TMC4. A Representative current–voltage (I-V) relationship of the currents by step pulses with KCl (left) or N-Methyl-d-glucamine (NMDG) -Cl (right) pipette solution in human embryonic kidney (HEK) 293 T cells expressing human TMC4 (hTMC4). B Representative I–V relationship of the currents by step pulses under different bath solutions in cation. The current observed is not affected by the bath application of NMDG-Cl, NaCl, or amiloride (epithelial sodium channel inhibitor), but is significantly reduced by the anion channel inhibitor, 5-nitro-2-(3-phenylpropylamino) benzoic acid (NPPB). Bath and pipette solution components and calculation procedu [file 12576_2021_807_MOESM1_ESM.pdf]

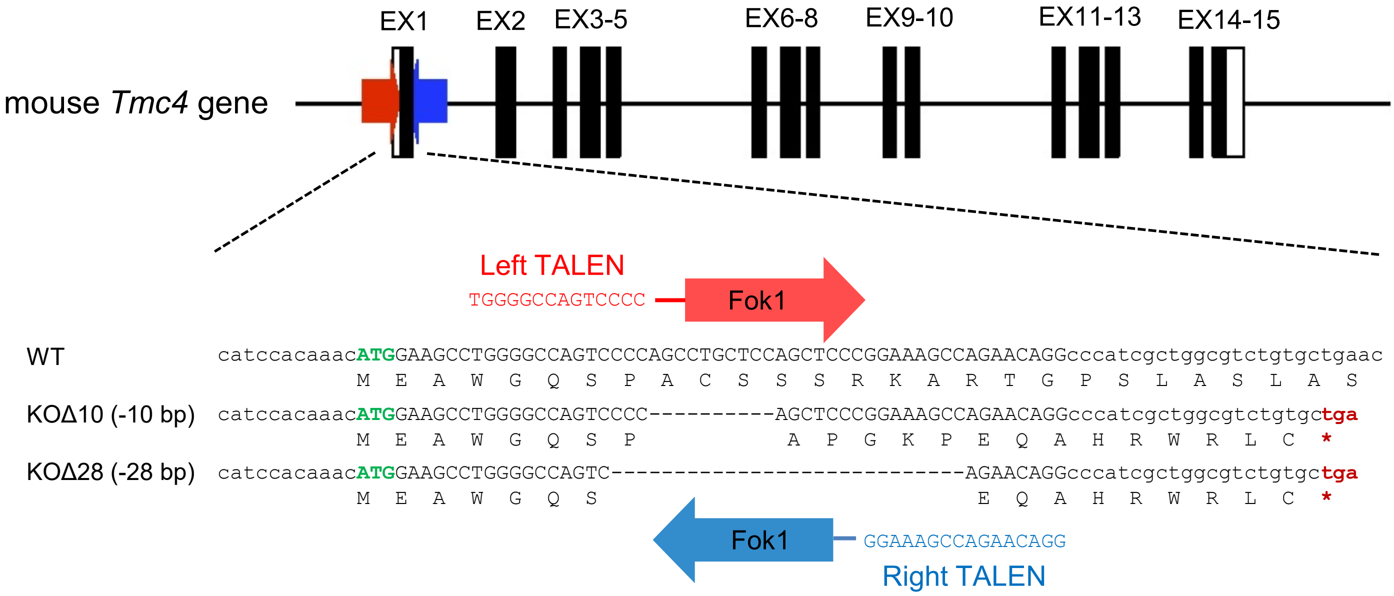

EX1
EX2
EX3-5
EX6-8
EX9-10
EX11-13
EX14-15
mouse Tmc4 gene
Left TALEN
TGGGGCCAGTCCCC
Fok1
WT
catccacaaacATGGAAGCCTGGGGCCAGTCCCCAGCCTGCTCCAGCTCCCGGAAAGCCAGAACAGGcccatcgctggcgtctgtgctgaac
M E A W G Q S P A C S S S R K A R T G P S L A S L A S
KOΔ10 (-10 bp)
catccacaaacATGGAAGCCTGGGGCCAGTCCCC----------AGCTCCCGGAAAGCCAGAACAGGcccatcgctggcgtctgtgctga
M E A W G Q S P A P G K P E Q A H R W R L C *
KOΔ28 (-28 bp)
catccacaaacATGGAAGCCTGGGGCCAGTC----------------------------AGAACAGGcccatcgctggcgtctgtgctga
M E A W G Q S E Q A H R W R L C *
Fok1
GGAAAGCCAGAACAGG
Right TALEN

Figure S1

A

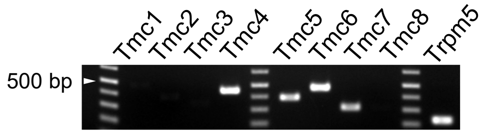

B

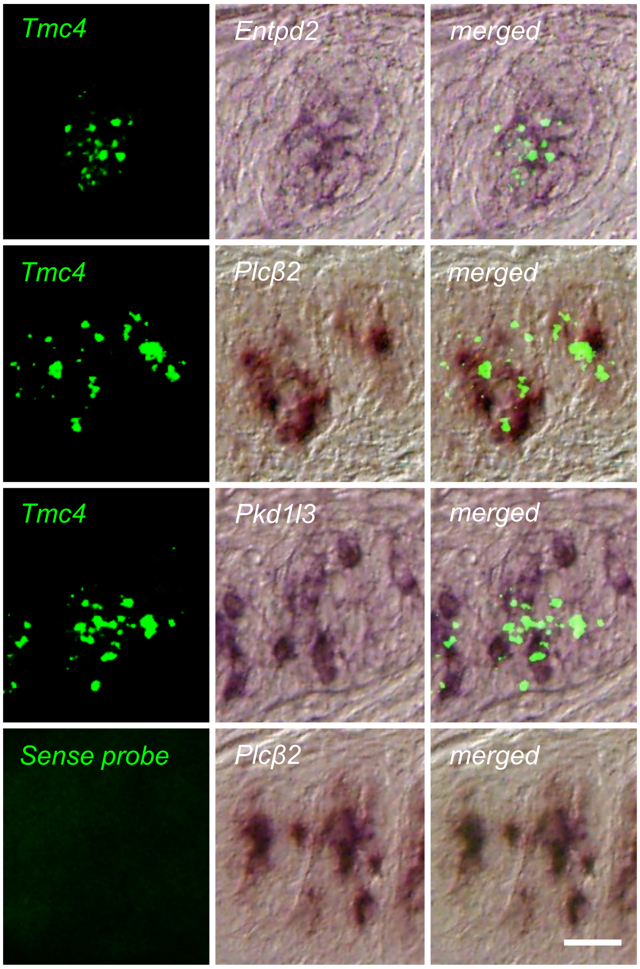

Scale bar 20 μm

Figure S2

hTMC4

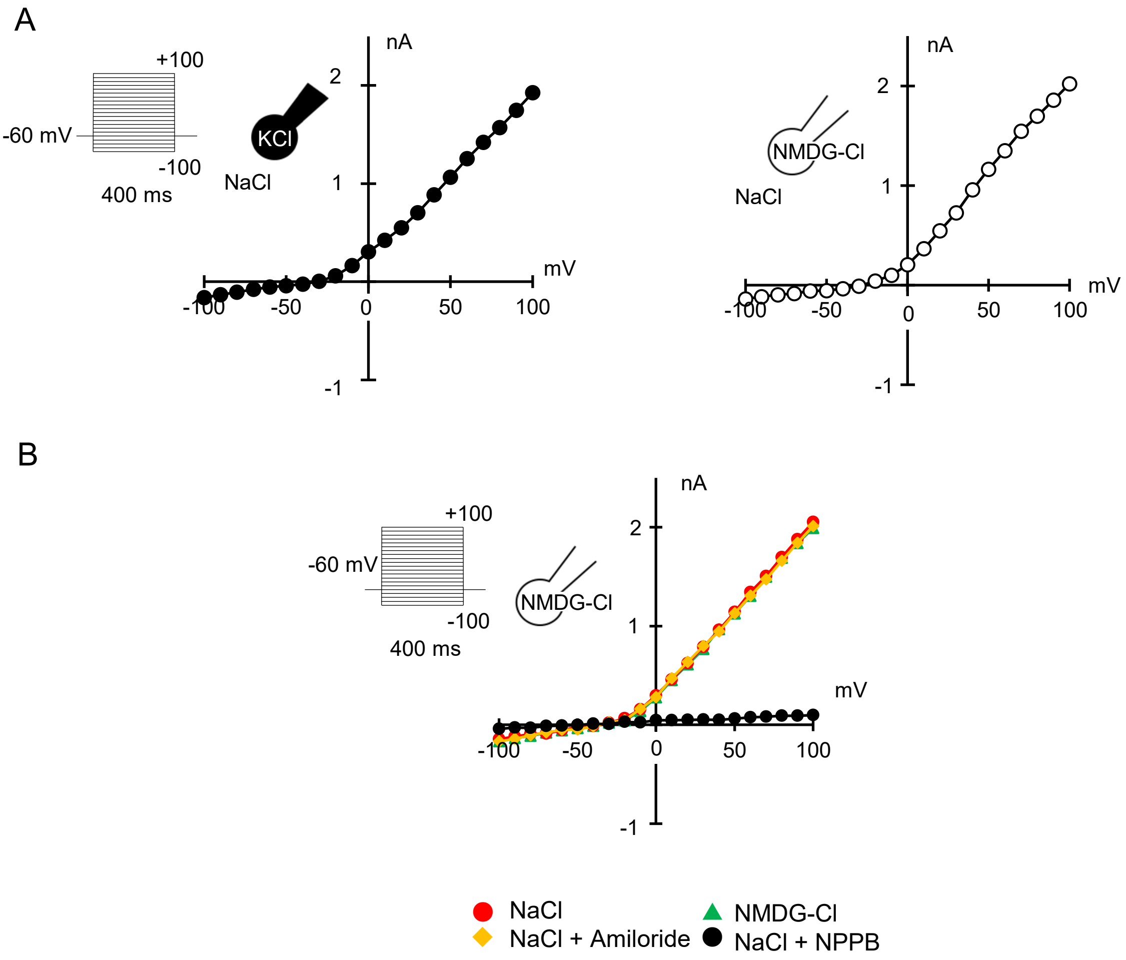

Figure S3

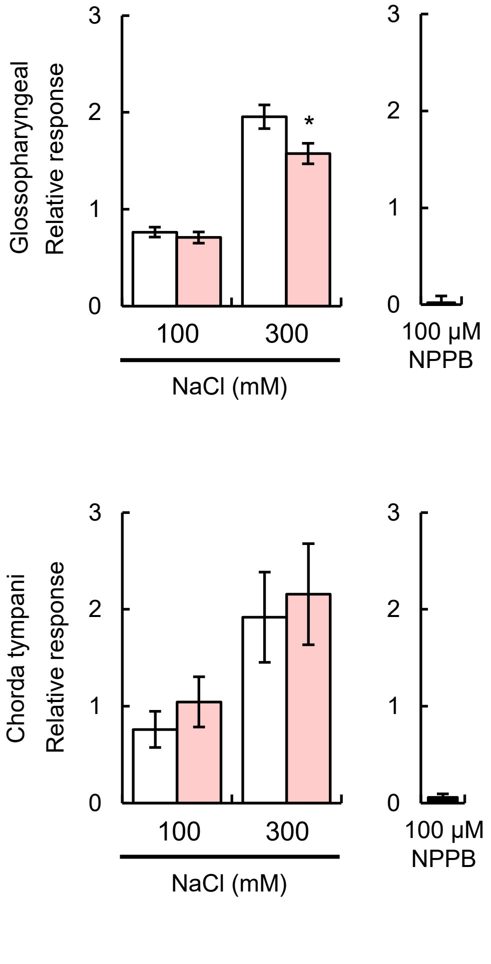

Glossopharyngeal
Relative response
3
2
1
0
*
100
300
NaCl (mM)
3
2
1
0
100 μM
NPPB
Chorda tympani
Relative response
3
2
1
0
100
300
NaCl (mM)
3
2
1
0
100 μM
NPPB
NaCl alone,
NaCl + 100 μM NPPB

Figure S4

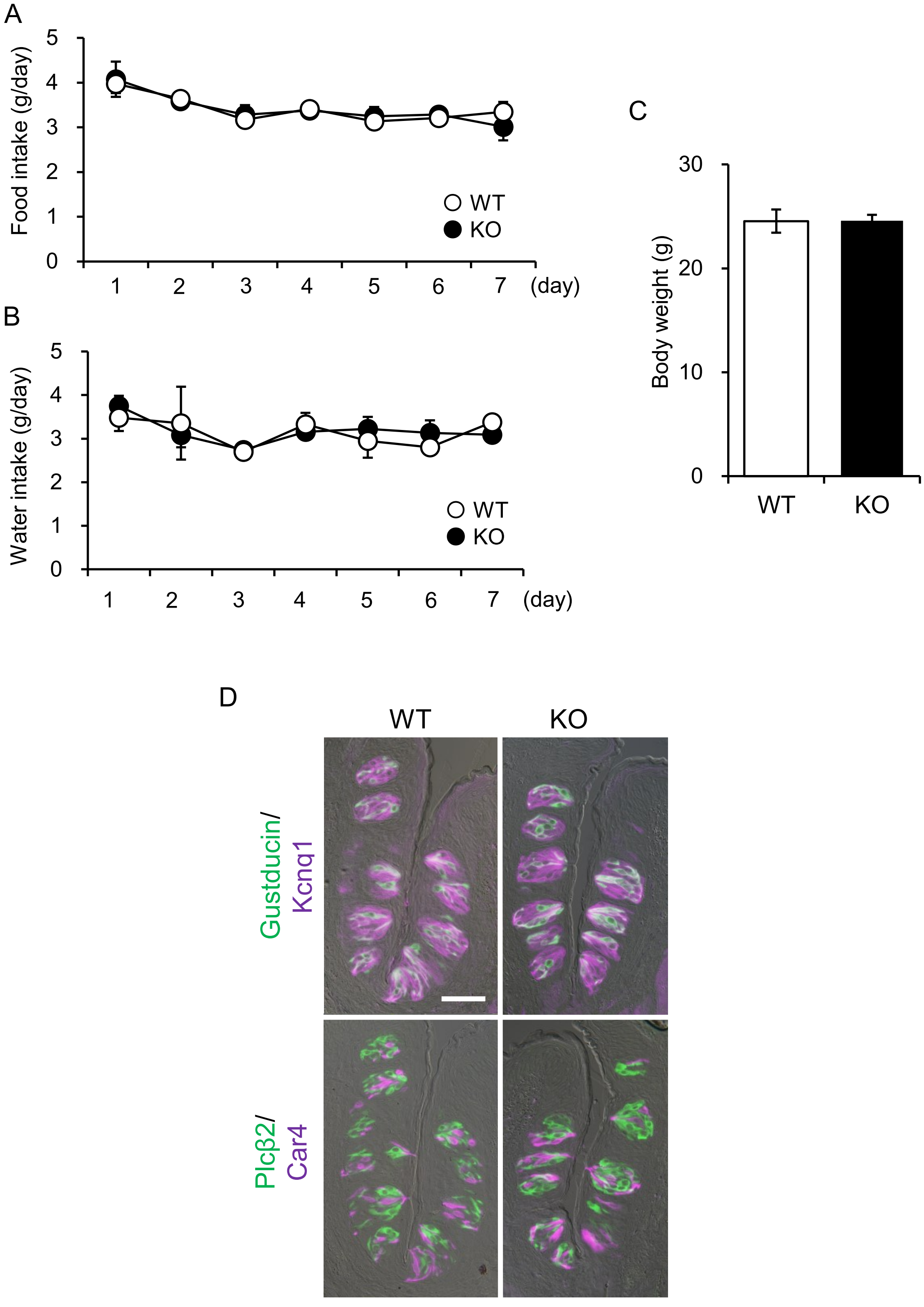
A
Food intake (g/day)
5
4
3
2
1
0
WT
KO
1
2
3
4
5
6
7
(day)
B
Water intake (g/day)
C
30
20
10
Body weight (g)
D
Gustducin/
Kcnq1
Plcβ2/
Car4

Figure S5

Table S1.
The compositions of pipette solutions

Electrolyte (mM) \ Solution	NMDG-Cl	KCl
NMDG-Cl	134	-
KCl	-	134
BAPTA	5	5
HEPES	10	10

N-Methyl-D-glucamine (NMDG), *O,O*'-Bis(2-aminophenyl)ethyleneglycol-*N,N,N',N'*-tetraacetic acid, tetrapotassium salt, hydrate (BAPTA), 4-(2-hydroxyethyl)-1-piperazineethanesulfonic acid (HEPES). The pH of all pipette solution was adjusted to pH 7.2 with NMDG-OH. The osmotic pressure of all pipette solution was adjusted to approximately 270 mOsmol/kg.

Table S2.
The compositions of bath solutions

Solution / Electrolyte (mM)	NaCl (Cintrol)	NMDG-Cl	67 mM NaCl, 67 mM Na-gluconate	34 mM NaCl, 34 mM Na-gluconate	Na-gluconate	NPPB	Na-glutamate	Asp-Na	Ionomycin
NaCl	134	-	67	34	-	134	-	-	134
Na-gluconate	-	-	67	100	134	-	-	-	-
KCl	2.9	2.9	2.9	2.9	2.9	2.9	2.9	2.9	2.9
HEPES	10	10	10	10	10	10	10	10	10
D-Glucose	15	15	15	15	15	15	15	15	15
$MgCl_2$	1.2	1.2	1.2	1.2	1.2	1.2	1.2	1.2	1.2
$CaCl_2$	2.1	2.1	2.1	2.1	2.1	2.1	2.1	2.1	2.1
NMDG-Cl	-	134	-	-	-	-	-	-	-
Na-glutamate	-	-	-	-	-	-	134	-	-
Asp-Na	-	-	-	-	-	-	-	134	-
NPPB	-	-	-	-	-	0.1	-	-	-
Ionomycin	-	-	-	-	-	-	-	-	0.005

N-Methyl-D-glucamine (NMDG), 4-(2-hydroxyethyl)-1-piperazineethanesulfonic acid (HEPES), 5-Nitro-2-(3-phenylpropylamino) benzoic acid (NPPB). The pH of all bath solution was adjusted to pH 7.4 with NMDG-OH. The osmotic pressure of all bath solution was adjusted to approximately 295 mOsmol/kg.

Table S3.
Comparison of serum and urine parameters

		WT	Tmc4 KO
Serum	ALB (g/dL)	3.20 ± 0.07	3.15 ± 0.06
	CRE (mg/dL)	0.13 ± 0.01	0.14 ± 0.01
	UA (mg/dL)	3.26 ± 0.09	3.12 ± 0.45
	UA/CRE	25.9 ± 1.27	23.7 ± 4.14
	Na (mEq/L)	148 ± 0.79	148 ± 0.58
	K (mEq/L)	9.72 ± 0.46	10.1 ± 0.29
	Cl (mEq/L)	106 ± 0.37	105 ± 0.60
	Ca (mg/dL)	9.66 ± 0.14	9.80 ± 0.15
	P (mg/dL)	9.90 ± 1.30	10.8 ± 1.21
	Mg (mg/dL)	4.08 ± 0.13	4.32 ± 0.18
	Fe (µg/dL)	125 ± 17.0	164 ± 14.9
24h urine	weight (g)	1.62 ± 0.30	1.53 ±0.15
	CRE (mg/dL)	32.3 ± 2.56	35.3 ± 2.32
	ALB/CRE	0.05 ± 0.00	0.04 ± 0.00
	TP/CRE	10.8 ± 3.45	11.4 ± 1.28
	BNU/CRE	123 ± 7.23	119 ± 2.52
	UA/CRE	0.78 ± 0.35	0.24 ± 0.02
	Na (mEq/L)	99.3 ± 8.79	110 ± 10.2
	K (mEq/L)	380.2 ± 33.1	389 ± 21.5
	Cl (mEq/L)	173 ± 17.0	192 ± 10.3
	Ca (mg/dL)	8.88 ± 1.89	6.87 ± 1.31
	P (mg/dL)	125 ± 22.4	114 ± 15.7
	Mg (mg/dL)	77.5 ± 15.7	79.1 ± 8.77

Some urine data were shown in ratio to creatine levels. No significant difference in all items using *t*-test (WT, KO: n = 6 for each). Abbreviations: WT; wild type, KO; knock-out, ALB; albumin, CRE; creatine, UA; uric acid, TP; total protein, BNU; blood urea nitrogen.

Table S4.
Property of salt responsive cells

	Amiloride sensitivity	NaCl		KCl	NMDG-Cl	Na-gluconate	Anion dependency
		100 mM	500 mM	500 mM	500 mM	250 mM	
HIgh salt cell	−	−	+	+	+	weak	+
	−	−	+	+	+	+	−
Low salt cell	+	+	+	−	−	+	−

Plus (+) and minus (-) indicate responsive and not responsive respectively. Weak means that the response of 250 mM Na-gluconate is weaker than that of NaCl.
