# Supplementary material for: TMC4 is a novel chloride channel involved in high-concentration salt taste sensation
Source: J Physiol Sci. 2021 Aug 25;71:23. doi: 10.1186/s12576-021-00807-z (PMC10717410; doi:10.1186/s12576-021-00807-z)
Supplement: Supplementary file 2 — Additional file 2. Description of the Mathematical Model of a Taste Bud Cell. [file 12576_2021_807_MOESM2_ESM.pdf]

Additional File2

Description of the Mathematical Model of a Taste Bud Cell

This supplemental data describe details of the mathematical model of a taste bud cell. Mathematical formulations are described in Tables S1-S4. The basic frame of the model was adapted from the model by Kimura *et al.*, 2014 (Kimura model). Kimura model utilized constant reversal potential values (E_{Na}, E_K, and E_{Cl}), which we updated so that they vary according to the intracellular and extracellular ionic concentrations (Table S2). A putative Cl^- current (TEA-insensitive current; I_{TI}) model in Kimura model was substituted by a newly constructed TMC4-mediated Cl^- current (I_{TMC4}) model (Figure S1).

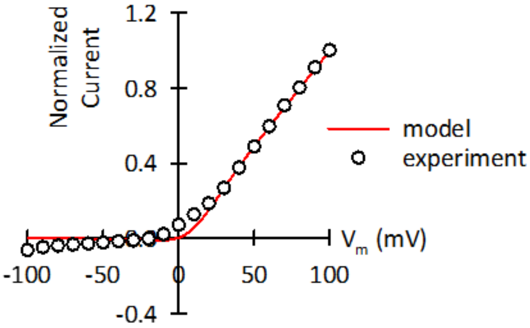

Figure S1. Current-voltage relationships of I_{TMC4}. A line is a model data and circles are experimental data from Figure 2A. The membrane potential was held at -60 mV, and test pulses of 400 ms duration were applied from -100 to +100 mV with 10 mV step every 1 s. Data are normalized to the current at +100 mV. $[Cl^-]_o$ = 143.5 mM, $[Cl^-]_i$ = 134 mM.

Parameters for several equations were modified as follows to better reproduce experimental data obtained using Type II taste bud cells (Medler *et al.*, 2003; Kimura *et al.*, 2014; Ma *et al.*, 2017). In Kimura model, delay in voltage-dependent Na^+ current (I_{Na}) activation was neglected; i.e., $m^x = m_\infty(V_m)$. In the present study, we employed activation kinetics of I_{Na} in Hodgkin-Huxley model (Hodgkin and Huxley, 1952). The time course of I_{Na} in voltage-clamp experiment well corresponds to the experimental data in Kimura *et al.*, 2014 (Figure S2). The maximum current densities of I_{Na} are −141.5 and −170.9 pA/pF with experimental condition of Ma *et al.*, 2017 and Kimura *et al.*, 2014, respectively, which are within the range of reported values in Type II cells (Kimura *et al.*, 2014; Ma *et al.*, 2017).

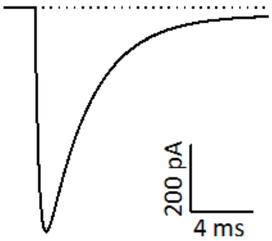

Figure S2. Time course of I_{Na} in voltage-clamp protocol. I_{Na} was induced with a test potential of -20 mV from a holding potential of -70 mV. A dotted line represents 0 pA. $[Na^+]_o$ = 150 mM, $[Na^+]_i$ = 10.9 mM.

The experimentally measured current density of a putative K^+ current (TEA-sensitive current; I_{TS}) was ~45 pA/pF and the ratio of I_{TS} to total outward current amplitude was ~20% in Kimura *et al.*, 2014. In the present study, we set conductances of I_{TS} and I_{TMC4} so that amplitudes of the corresponding currents become within the experimental data.

The new model cell was created with a Visual C# (Microsoft Visual Studio 2019) and the ordinary differential equations were integrated with the fourth order Runge-Kutta method with adaptive time step. Units are pA for current, mV for membrane potential, and ms for time. The resting membrane potential of the model cell is −58.2 mV, which is within the range of experimental data (Medler *et al.*, 2003; Kimura *et al.*, 2014; Ma *et al.*, 2017). When 10 pA holding current (I_{hold}) is applied, the resting membrane potential becomes −70.1 mV, well reproducing experimental data by Ma *et al.*, 2017. Configurations of whole cell currents obtained by voltage-clamped condition well correspond to experimental data by both Kimura *et al.*, 2014 and Ma *et al.*, 2017 (Figures S3).

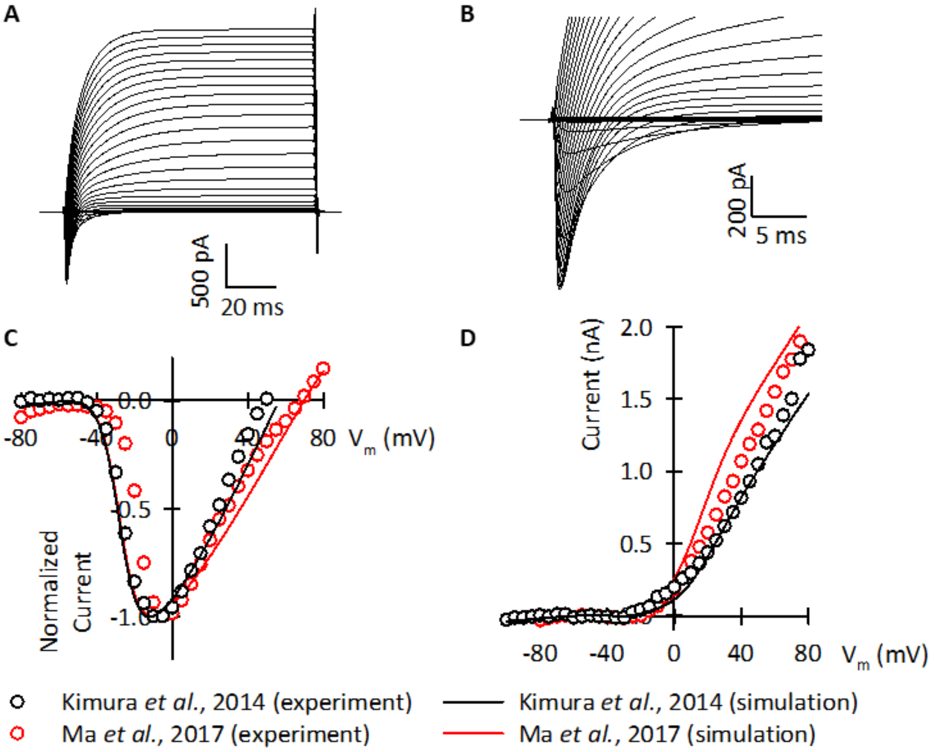

Figure S3. Simulation of membrane current of a taste bud cell. **A, B.** Total membrane currents evoked by 100 ms voltage pulses of -80 mV to +80 mV from a holding potential of -70 mV. Enlarged traces focusing on inward currents are shown in **B**. **C, D.** Current-voltage relationships of peak inward currents (**C**) and steady state outward currents (**D**) compared with experimental data. Lines are model data and circles are experimental data from Kimura *et al.*, 2014 (black) and Ma *et al.*, 2017 (red). $[Na^+]_o$ = 150 mM, $[K^+]_o$ = 5 mM, $[Cl^-]_o$ = 160 mM, $[Na^+]_i$ = 10.9 mM, $[K^+]_i$ = 150 mM, $[Cl^-]_i$ = 134.8 mM for Kimura *et al.*, 2014 condition. $[Na^+]_o$ = 150 mM, $[K^+]_o$ = 5.4 mM, $[Cl^-]_o$ = 150 mM, $[Na^+]_i$ = 6 mM, $[K^+]_i$ = 140 mM, $[Cl^-]_i$ = 30 mM for Ma *et al.*, 2017 condition.

In addition, single action potential configuration with the dV_m/dt, as well as stimulation current (I_{stim}) amplitude-dependent generations of action potentials reported by Ma *et al.*, 2017 are beautifully reproduced by the model (Figure 4A and Figure S4).

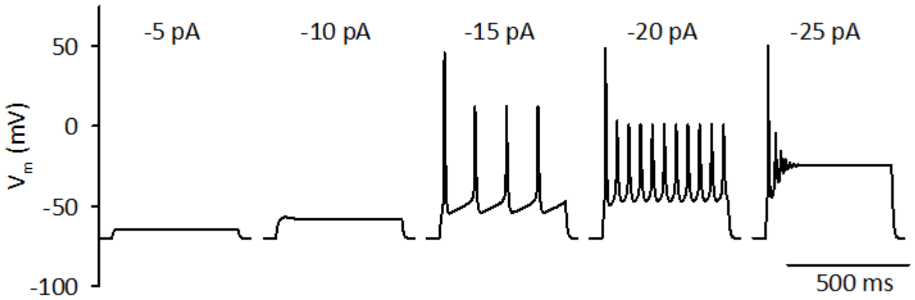

Figure S4. Trains of action potentials evoked by various I_{stim}. The model cell was held at -70 mV with 10 pA I_{hold}. $[Na^+]_o$ = 150 mM, $[K^+]_o$ = 5.4 mM, $[Cl^-]_o$ = 150 mM, $[Na^+]_i$ = 6 mM, $[K^+]_i$ = 140 mM, $[Cl^-]_i$ = 30 mM.

Table S1. Abbreviations

R	gas constant, 8.3143 C mV/K/mmol
F	Faraday's constant, 96.4867 C/mmol
T	absolute temperature, 310 K
$[Na^+]_o$	extracellular Na^+ concentration
$[Na^+]_i$	cytoplasmic Na^+ concentration
$[K^+]_o$	extracellular K^+ concentration
$[K^+]_i$	cytoplasmic K^+ concentration
$[Cl^-]_o$	extracellular Cl^- concentration
$[Cl^-]_i$	cytoplasmic Cl^- concentration
C_m	cell capacitance, 5.0 pF, adapted from Ma *et al.*, 2017

Table S2. Reversal potentials of ions

$$E_{Na} = \frac{RT}{F} \ln \frac{[Na^+]_o}{[Na^+]_i}$$

$$E_K = \frac{RT}{F} \ln \frac{[K^+]_o}{[K^+]_i}$$

$$E_{Cl} = -\frac{RT}{F} \ln \frac{[Cl^-]_o}{[Cl^-]_i}$$

Table S3. Membrane potential

$$\frac{dV_m}{dt} = -\frac{1}{C_m}(I_{Na} + I_{TS} + I_{Tmc4} + I_L + I_{hold} + I_{stim})$$

I_{hold}; holding current, I_{stim}; stimulation current

Table S4. Ionic currents

I_{Na}; Voltage-dependent Na^+ current

$$m_{inf} = \frac{1.0}{1.0 + \exp\left(\frac{-(V_m + 26.0)}{5.0}\right)}$$

$$\tau_m = \frac{1.0}{\frac{-0.1 \cdot (V_m + 50.0)}{\exp\left(-0.1 \cdot (V_m + 50.0)\right) - 1.0} + 4.0 \cdot \exp\left(\frac{-(V_m + 75.0)}{18.0}\right)}$$

$$h_{inf} = \frac{1.0}{1.0 + \exp\left(\frac{V_m + 60.0}{10.0}\right)}$$

$$\tau_h = \frac{1272}{13.6 \cdot \sqrt{2\pi}} \cdot \exp\left(\frac{-(V_m + 59.0)^2}{2 \cdot 13.6^2}\right) + 2.2$$

$$\frac{dm}{dt} = \frac{m_{inf} - m}{\tau_m}$$

$$\frac{dh}{dt} = \frac{h_{inf} - h}{\tau_h}$$

$$I_{Na} = g_{Na} \cdot m \cdot h \cdot \ (V_m - E_{Na});\ \ g_{Na} = 19.1 \text{ nS}$$

I_{TS}; Delayed rectifier K^+ current (TEA-sensitive current)

$$n_{inf} = \frac{1.0}{1.0 + \exp\left(\frac{-(V_m - 2.3)}{11.0}\right)}$$

$$\tau_n = 4.7 \cdot \exp\left(\frac{-(V_m + 23.7)^2}{2500}\right) + 1.1$$

$$\frac{dn}{dt} = \frac{n_{inf} - n}{\tau_n}$$

$$I_{TS} = g_{TS} \cdot n \cdot \ (V_m - E_K);\ \ g_{TS} = 0.9375 \text{ nS}$$

I_{TMC4}; TMC4-mediated Cl^- current

$$gate_{inf} = \frac{0.95}{1.0 + \exp\left(\frac{-(V_m - 10.0)}{10.0}\right)} + \frac{0.05}{1.0 + \exp\left(\frac{-(V_m - 60.0)}{28.0}\right)}$$

$$\tau_{gate} = \frac{1.0}{\frac{0.0016 \cdot (V_m + 2.3)}{1.0 - \exp\left(\frac{-(V_m + 2.3)}{5.6}\right)} + \frac{23.0 \cdot (V_m + 180)}{\exp\left(\frac{V_m + 180}{15.9}\right) - 1.0} + 0.0066}$$

$$\frac{dgate}{dt} = \frac{gate_{inf} - gate}{\tau_{gate}}$$

$$I_{TMC4} = g_{Tmc4} \cdot gate \cdot (V_m - E_{Cl});\ \ g_{Tmc4} = 15.0\ \text{nS}$$

I_L; Leak current[1]

$$I_L = g_L \cdot (V_m - 60.5);\ \ g_L = 0.98\ \text{nS}$$

References

Hodgkin AL and Huxley AF (1952) A quantitative description of membrane current and its application to conduction and excitation in nerve. *J Physiol* 117(4):500-44

Kimura K, Ohtubo Y, Tateno K, Takeuchi K, Kumazawa T, Yoshii K (2014) Cell-type-dependent action potentials and voltage-gated currents in mouse fungiform taste buds. *Eur J Neurosci* 39(1):24-34

Ma Z, Saung WT, Foskett JK (2017) Action potentials and ion conductances in wild-type and CALHM1-knockout type II taste cells. *J Neurophysiol* 117(5):1865-1876

Medler KF, Margolskee RF, Kinnamon SC (2003) Electrophysiological characterization of voltage-gated currents in defined taste cell types of mice. *J Neurosci* 23(7):2608-17
